# Supplementary material for: Exploring Salinity Tolerance Mechanisms in Diverse Wheat Genotypes Using Physiological, Anatomical, Agronomic and Gene Expression Analyses
Source: Plants (Basel). 2023 Sep 20;12(18):3330. doi: 10.3390/plants12183330 (PMC10535590; doi:10.3390/plants12183330)
Supplement: Supplementary file 1 [file plants-12-03330-s001.zip › plants-2600093-supplementary.pdf]

Table S1. Name, pedigree and origin of the studied bread wheat genotypes

Name	Code	Pedigree	Origin
Ismailia line	G1	Selected mutation	Egypt
Misr 1	G2	OASIS / SKAUZ // 4*BCN /3/ 2*PASTOR CMSS00Y01881T-050M-030Y-030M-030WGY-33M-0Y-0S	Egypt
Misr 3	G3	ATTILA*2/PBW65*2 /KACHU	Egypt
